# Increased Gene Expression in Cultured BEAS-2B Cells Treated with Metal Oxide Nanoparticles

**DOI:** 10.5487/TR.2009.25.4.195

**Published:** 2009-12-30

**Authors:** Eun-Jung Park, Kwangsik Park

**Affiliations:** College of Pharmacy, Dongduk Women’s University, 23-1, Wolgok-dong, Seongbuk-gu, Seoul, 136-714 Korea

**Keywords:** Cerium oxide nanoparticles, Titanium oxide nanoparticles, Microarray, Gene expression

## Abstract

Recent publications showed that metal nanoparticles which are made from TiO_2_, CeO_2_, Al_2_O_3_, CuCl_2_, AgNO_3_ and ZnO_2_ induced oxidative stress and pro-inflammatory effects in cultured cells and the responses seemed to be common toxic pathway of metal nanoparticles to the ultimate toxicity in animals as well as cellular level. In this study, we compared the gene expression induced by two different types of metal oxide nanoparticles, titanium dioxide nanoparticles (TNP) and cerium dioxide nanoparticles (CNP) using microarray analysis. About 50 genes including interleukin 6, interleukin 1, platelet-derived growth factor β, and leukemia inhibitory factor were induced in cultured BEAS-2B cells treated with TNP 40 ppm. When we compared the induction levels of genes in TNP-treated cells to those in CNP-treated cells, the induction levels were very correlated in various gene categories (r = 0.645). This may suggest a possible common toxic mechanism of metal oxide nanoparticles.

## NTRODUCTION

The discharge of nanomaterials to environment could be rapidly increased with the increased application of nanomaterials and it may exhibit adverse effects to human health. Titanium dioxide nanoparticles (TNP) have been widely used as a white pigment in paint, food colorant, ultraviolet blocker in cosmetics, welding rod-coating material, disinfectant in environment and wastewater, and photosensitizer for photodynamic therapy (Gurr *et al.*, 2005). Cerium dioxide nanoparticles (CNP) have been used for solar cells, fuel cells, gas sensors, oxygen pumps, and glass/ceramic applications (Gao *et al.*, 2006). Exposure to TNP was known to induce cytotoxicity, oxidative stress, lung inflammation, cell proliferation, and histopathological responses, with differential pulmonary effects (Warheit *et al.*, 2007; Park *et al.*, 2008a, b; Reeves *et al.*, 2008; Kang *et al.*, 2008). It increased the production of hydrogen peroxide and nitric oxide in human bronchial epithelial cells (Gurr *et al.*, 2005) and also caused apoptosis in Syrian hamster embryo fibroblasts (Rahman *et al.*, 2002).

Regarding the toxicity of CNP, it also induced reactive oxygen species (ROS) and cytotoxicity in A549 cells. The elevated oxidative stress was found to increase the production of malondialdehyde which is the indicator of lipid peroxidation and membrane damage (Lin *et al.*, 2006). CNP also showed apoptotic process in cultured BEAS-2B cells which are derived from human bronchoalveolar epithelial cells with increased ROS (Park *et al.*, 2008b), which shows similarity with TNP in generating toxic responses. Furthermore, many publications showed the oxidative stress and pro-inflammatory responses were induced when the cultured cells were treated with nanomaterials prepared from metals such as silver, zinc and copper (Carlson *et al.*, 2008; Karlsson *et al.*, 2008; Rahman *et al.*, 2009; Sharma *et al.*, 2009).

The focus of the present study is to evaluate and compare the gene expressions increased in TNP- and CNP-treated cells, which may suggest that metal nanoparticles may have a common pathway to exert toxicological responses.

## MATERIALS AND METHODS

***Metal oxide nanoparticles.*** Commercial titanium dioxide nanoparticles (TNP, P-25, 21 nm-size) were purchased from Degussa Korea (Incheon, Korea). According to the information provided by the supplier, titanium dioxide P25 is a fine white powder with hydrophilic character caused by hydroxyl groups on the surface (ca. 5 OH-groups/nm$^2$). Cerium oxide nanoparticles (CNP, 30 nm) were prepared by the supercritical synthesis method as previously reported (Park *et al.*, 2008b). The suspensions of CNP and TNP were sonicated prior to test due to aggregation. For cytokine assay, cells were treated with CNP and TNP for 24 hours (5, 10, 20, 40 ppm).

***Physico-chemical analysis.*** Particle sizes in test media (DMEM/F12 medium, 10% FBS) were measured before experiment using a submicron particle sizer (NICOMP$^{TM}$, CA, USA), and images of the nanoparticles were acquired by transmission electron microscopy (TEM; JEM1010, JEOL, Japan). The surface charges of TNP and CNP suspended in distilled water were also measured using Zeta potential analyzer (Brookhaven instruments corp., NY, USA), respectively.

***Cell culture and RNA preparation.*** BEAS-2B cells were maintained in DMEM/F12 medium (GIBCO Invitrogen, Seoul, Korea) containing 10% FBS, penicillin 100 IU/ml, and streptomycin 100 μg/ml, respectively. Cells were grown and maintained in 28 cm$^2$ cell culture flasks at 37°C in a 5% $CO_2$ humidified incubator. For the preparation of total RNA, cells were treated for 4 hours with titania and ceria of 40 ppm, respectively, and the RNAgent Total RNA Isolation System (Promega, Madison, WI, USA) was used according to the manufacturer's instructions.

***Microarray analysis.*** Microarray analysis was performed by macrogen using Applied Biosystems Human Genome Survey. Briefly, digoxigenin-11-UTP labeled cRNA was generated and linearly amplified from 1 μg of total RNA purified from control and nanoparticles exposure group, respectively, using Applied Biosystems Chemiluminescent RT-IVT Labeling Kit. Array hybridization, chemiluminescence detection, image acquisition and analysis were performed using Applied Biosystems Chemiluminescence Detection Kit and Applied Biosystems 1700 Chemiluminescent Microarray Analyzer following manufacturer's protocol. Each image was collected for each microarray using the 1700 analyzer equipped with high-resolution, large-format CCD camera for gene expression analysis. Images were autogridded and the chemiluminescent signals were quantified, corrected for background and spot, and spatially normalized.

***Measurement of cytokines.*** The concentration of IL-6 and IL-8 secreted from the cultured BEAS-2B cells was determined using ELISA kits commercially available from eBioscience (San diego, CA, USA). Briefly, microplates were coated with 100 μl of capture antibody per well and incubated overnight at 4°C. After washing and blocking with assay diluent, the supernatant of cell and standards were added to each well and the plates were maintained for 2 hours at room temperature (RT). The plates were washed and biotin-conjugated detecting mouse antibody was added to each well and incubated at RT for 1 hour. The plate further incubated with avidin-HRP for 30 min before detection using TMB solution. Finally, response was stopped with 1 M $H_3PO_4$, and absorbance was measured at 450 nm with an ELISA reader (Molecular Devices, Sunnyvale, CA, USA). The amounts of cytokines were calculated from the linear portion of the standard curve.

## RESULTS AND DISCUSSION

***Physico-chemical properties of TNP and CNP.*** Surface charge and size-distribution of nanoparticles are very important parameters in toxicity test. Some report described that smaller particles have more toxic to cells and the agglomeration/aggregation of particles may affect the toxicity (Wittmaack, 2007).

The surface charges of TNP and CNP suspended in distilled water were 18.66 mV and 43.44 mV, respectively (Fig. 1A) when detected using Zeta-potential analyzer. However, those were changed to - 13.73 mV and -1.21 mV in cultured media (Fig. 1B) (DMEM/F12, 10% FBS). The decrease of surface charge means that the particles may be agglomerated or aggregated. The decreased levels of surface charges in TNP and CNP were very similar, which means that the agglomeration level was also very similar. The particle sizes of TNP and CNP were 21 nm and 30 nm when they were prepared and maintained in dried-powder form. However, the particles were agglomerated or aggregated when they were suspended in culture media. The distribution of particles size was shown in Fig. 2. Average diameter of TNP in culture media was increased 134.6 nm and CNP was increased 105.6 nm. TEM images of TNP and CNP are shown in Fig. 3. According to the TEM images of the nanoparticles, they seem to be attached through weak bindings.

***Gene expression.*** Table 1 shows the partial list of genes which was upregulated in cultured BEAS-2B cells treated with TNP and CNP, respectively. Results were represented as an average value of two separate experiments. According to the ontology analysis, genes related with signal transduction, immune reaction

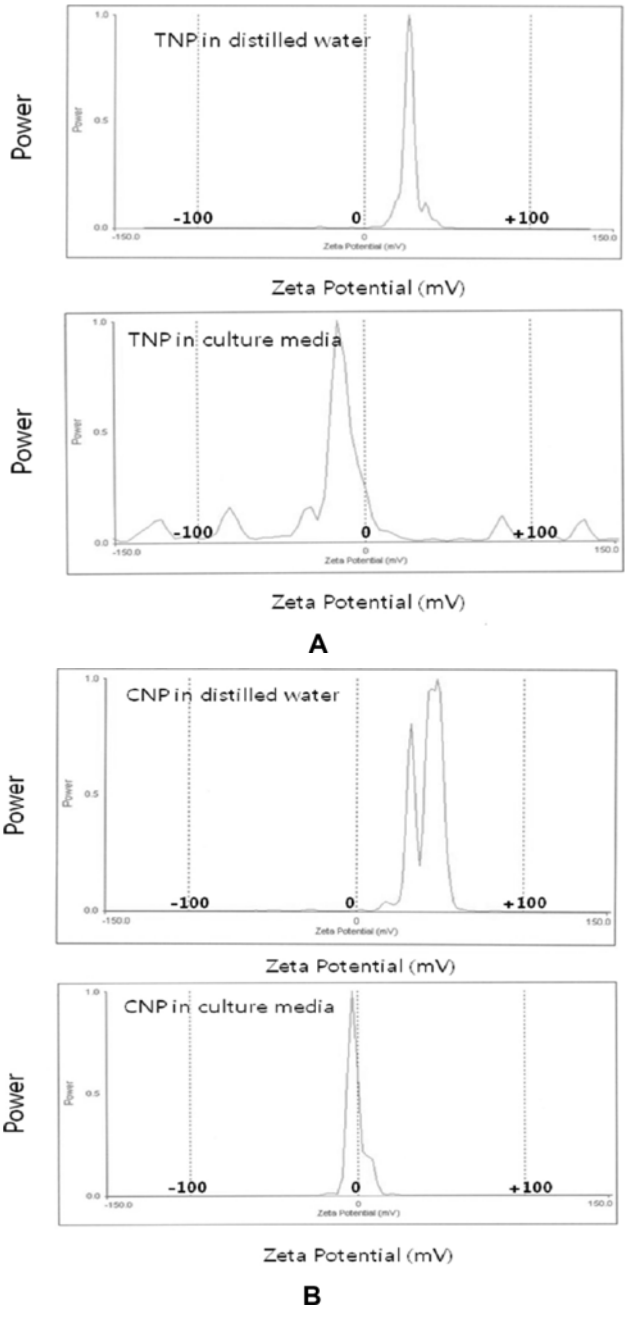

**Fig. 1.** Surface charges of TNP and CNP measured by Zeta Potential. **A**: TNP 40 ppm was suspended in distilled water (upper) and in DMEM/F12 medium with 10% FBS. **B**: CNP 40 ppm was suspended in distilled water (lower) and in DMEM/F12 medium with 10% FBS. The decreased zeta potential means the agglomeration of nanoparticles.

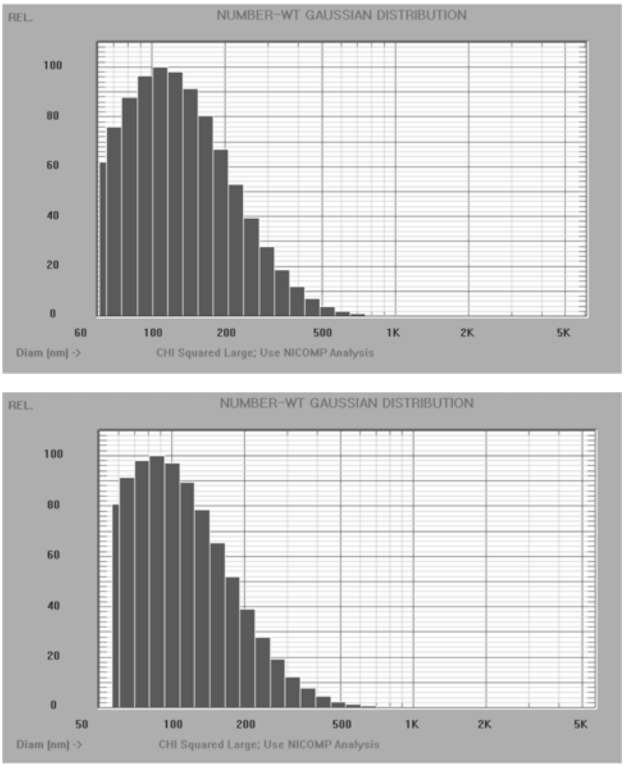

**Fig. 2.** Size-distribution of TNP and CNP in culture media. TNP 40 ppm and CNP 40 ppm were suspended in culture media, respectively and size-distribution was analyzed using a submicro particle sizer. Average sizes of TNP (upper panel) CNP (lower panel) were 134.6 nm and 105.6 nm, respectively.

(inflammation), apoptosis, and nucleic acid metabolism were mainly upregulated. Among them, upregulation of genes related to immunity system (inflammation) including leukemia inhibitory factor, interleukin 6, interleukin 1, tumor necrosis factor, CXC chemokine family and platelet-derived growth factor were most distinguished. The induction of the inflammatory genes was coincided with previous reports on the pro-inflammatory responses of TNP in *in vivo* studies (Park *et al.*, 2009a). Induction of inflammatory responses are also observed in silica nanoparticles and multi-walled carbon nanotubes (Park *et al.*, 2009, c).

Total number of genes induced by TNP in cultured BEAS-2B cells was not large. It was confirmed by separate experiments. Furthermore, down-regulated genes were not so many (data not shown). This phenomenon was similarly occurred in CNP-treated cells. The total number of induced genes in CNP-treated cells was not large like in TNP-treated cells. The reason why only a few genes are induced in metal nanoparaticles-treated cells is not understood at the present and this phenomenon is very unusual. When cultured cells are treated with metal nanoparticles, it seems that the particles penetrated cell membrane and accumulated in the nuclear membrane area (Park *et al.*, 2008b). The accumulation of the nanoparticles in the nuclear membrane area may block the nuclear pore to inhibit the free passage of

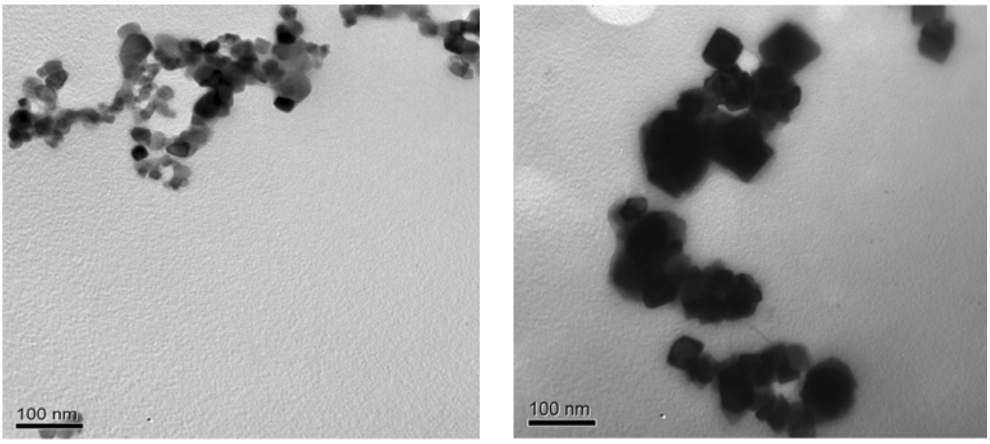

**Fig. 3.** TEM images of TNP and CNP in culture media. TNP and CNP were suspended in culture media and TEM images were obtained. Single particles seemed to be agglomerated and the agglomerated sizes of TNP (left panel) and CNP (right panel) are almost same with the data obtained by particle sizer shown in Fig. 2.

**Table 1.** Partial list of genes induced by titanium dioxide nanoparticles (TNP) and cerium dioxide nanoparticles (CNP)

| Symbol | Definition | TNP | | CNP | | Accession |
|---|---|---|---|---|---|---|
| | | Fold | SD | Fold | SD | |
| FOSB | FBJ murine osteosarcoma viral oncogene homolog B (FOSB), mRNA. | 6.26 | 0.68 | 4.11 | 0.86 | NM_006732.1 |
| ANGPTL4 | angiopoietin-like 4 (ANGPTL4), transcript variant 1, mRNA. | 5.50 | 0.60 | 3.94 | 0.15 | NM_139314.1 |
| FOS | v-fos FBJ murine osteosarcoma viral oncogene homolog (FOS), mRNA. | 5.32 | 0.00 | 2.70 | 0.22 | NM_005252.2 |
| EGR1 | early growth response 1 (EGR1), mRNA. | 4.63 | 0.05 | 2.00 | 0.48 | NM_001964.2 |
| PDGFB | platelet-derived growth factor beta polypeptide (simian sarcoma viral (v-sis) oncogene homolog) (PDGFB), transcript variant 2, mRNA. | 3.94 | 0.30 | 2.22 | 0.23 | NM_033016.1 |
| LIF | leukemia inhibitory factor (cholinergic differentiation factor) (LIF), mRNA. | 3.84 | 0.44 | 2.26 | 0.34 | NM_002309.2 |
| CSF2 | colony stimulating factor 2 (granulocyte-macrophage) (CSF2), mRNA. | 3.80 | 0.15 | 2.06 | 0.19 | NM_000758.2 |
| IL6 | interleukin 6 (interferon, beta 2) (IL6), mRNA. | 3.64 | 0.63 | 2.57 | 0.44 | NM_000600.1 |
| HBEGF | heparin-binding EGF-like growth factor (HBEGF), mRNA. | 3.22 | 0.15 | 2.12 | 0.19 | NM_001945.1 |
| ATF3 | activating transcription factor 3 (ATF3), transcript variant 3, mRNA. | 3.15 | 0.14 | 2.08 | 0.18 | NM_001030287.1 |
| IL1A | interleukin 1, alpha (IL1A), mRNA. | 3.08 | 1.03 | 1.93 | 1.06 | NM_000575.3 |
| BHLHB2 | basic helix-loop-helix domain containing, class B, 2 (BHLHB2), mRNA. | 3.04 | 0.37 | 2.14 | 0.47 | NM_003670.1 |
| LFNG | lunatic fringe homolog (Drosophila) (LFNG), mRNA. | 2.97 | 0.16 | 2.15 | 0.44 | NM_002304.1 |
| CXCL2 | chemokine (C-X-C motif) ligand 2 (CXCL2), mRNA. | 2.82 | 0.84 | 1.67 | 0.55 | NM_002089.1 |
| TMEPAI | transmembrane, prostate androgen induced RNA (TMEPAI), transcript variant 2, mRNA. | 2.79 | 1.00 | 2.15 | 0.54 | NM_199169.1 |
| IL11 | interleukin 11 (IL11), mRNA. | 2.76 | 0.11 | 2.58 | 0.52 | NM_000641.2 |
| TMEPAI | transmembrane, prostate androgen induced RNA (TMEPAI), transcript variant 1, mRNA. | 2.67 | 0.81 | 1.93 | 0.45 | NM_020182.3 |
| PTHLH | parathyroid hormone-like hormone (PTHLH), transcript variant 4, mRNA. | 2.61 | 0.34 | 1.62 | 0.13 | NM_198966.1 |
| TNF | tumor necrosis factor (TNF superfamily, member 2) (TNF), mRNA. | 2.55 | 0.40 | 1.30 | 0.73 | NM_000594.2 |
| SGK | serum/glucocorticoid regulated kinase (SGK), mRNA. | 2.43 | 0.11 | 1.98 | 0.32 | NM_005627.2 |
| TGM2 | transglutaminase 2 (C polypeptide, protein-glutamine-gamma-glutamyltransferase) (TGM2), transcript variant 1, mRNA. | 2.43 | 0.37 | 1.90 | 1.42 | NM_004613.2 |
| DUSP5 | dual specificity phosphatase 5 (DUSP5), mRNA. | 2.41 | 0.33 | 2.08 | 0.65 | NM_004419.3 |
| TNFAIP3 | tumor necrosis factor, alpha-induced protein 3 (TNFAIP3), mRNA. | 2.38 | 0.44 | 1.67 | 0.08 | NM_006290.2 |
| KLF6 | Kruppel-like factor 6 (KLF6), transcript variant 1, mRNA. | 2.38 | 0.18 | 1.33 | 0.44 | NM_001008490.1 |
| AXUD1 | AXIN1 up-regulated 1 (AXUD1), mRNA. | 2.36 | 0.14 | 1.64 | 0.57 | NM_033027.2 |
| ZC3H12A | zinc finger CCCH-type containing 12A (ZC3H12A), mRNA. | 2.35 | 0.01 | 1.53 | 0.04 | NM_025079.1 |
| IER3 | immediate early response 3 (IER3), transcript variant long, mRNA. | 2.34 | 0.11 | 1.79 | 0.24 | NM_052815.1 |
| EFNB2 | ephrin-B2 (EFNB2), mRNA. | 2.34 | 0.07 | 1.53 | 0.14 | NM_004093.2 |
| PLAU | plasminogen activator, urokinase (PLAU), mRNA. | 2.32 | 0.45 | 1.94 | 0.17 | NM_002658.2 |
| KLF10 | Kruppel-like factor 10 (KLF10), transcript variant 1, mRNA. | 2.32 | 0.30 | 1.72 | 0.70 | NM_005655.1 |
| IL1B | interleukin 1, beta (IL1B), mRNA. | 2.29 | 1.01 | 1.57 | 0.79 | NM_000576.2 |

**Table 1.** Continued

| Symbol | Definition | TNP | | CNP | | Accession |
|---|---|---|---|---|---|---|
| | | Fold | SD | Fold | SD | |
| CLCF1 | cardiotrophin-like cytokine factor 1 (CLCF1), mRNA. | 2.27 | 0.05 | 1.55 | 0.28 | NM_013246.2 |
| GADD45A | growth arrest and DNA-damage-inducible, alpha (GADD45A), mRNA. | 2.26 | 0.28 | 1.48 | 0.07 | NM_001924.2 |
| HIVEP1 | human immunodeficiency virus type I enhancer binding protein 1 (HIVEP1), mRNA. | 2.24 | 0.02 | 1.69 | 0.14 | NM_002114.1 |
| SERPINE1 | serpin peptidase inhibitor, clade E (nexin, plasminogen activator inhibitor type 1), member 1 (SERPINE1), mRNA. | 2.23 | 0.03 | 1.81 | 0.15 | NM_000602.1 |
| KLF6 | Kruppel-like factor 6 (KLF6), transcript variant 2, mRNA. | 2.22 | 0.20 | 1.30 | 0.40 | NM_001300.4 |
| MSC | musculin (activated B-cell factor-1) (MSC), mRNA. | 2.20 | 0.07 | 1.92 | 0.22 | NM_005098.2 |
| ANGPTL4 | angiopoietin-like 4 (ANGPTL4), transcript variant 1, mRNA. | 2.18 | 0.46 | 1.78 | 0.08 | NM_139314.1 |
| IRAK2 | interleukin-1 receptor-associated kinase 2 (IRAK2), mRNA. | 2.15 | 0.44 | 1.55 | 0.38 | NM_001570.3 |
| HMOX1 | heme oxygenase (decycling) 1 (HMOX1), mRNA. | 2.15 | 0.74 | 2.17 | 0.10 | NM_002133.1 |
| F2RL1 | coagulation factor II (thrombin) receptor-like 1 (F2RL1), mRNA. | 2.13 | 0.29 | 1.40 | 0.75 | NM_005242.3 |
| EDN1 | endothelin 1 (EDN1), mRNA. | 2.13 | 0.65 | 2.23 | 0.82 | NM_001955.2 |
| KLF10 | Kruppel-like factor 10 (KLF10), transcript variant 1, mRNA. | 2.09 | 0.26 | 1.56 | 0.65 | NM_005655.1 |
| RARA | retinoic acid receptor, alpha (RARA), transcript variant 2, mRNA. | 2.07 | 0.05 | 1.49 | 0.19 | NM_001024809.2 |
| TRIM47 | tripartite motif-containing 47 (TRIM47), mRNA. | 2.05 | 0.03 | 1.33 | 0.36 | NM_033452.2 |
| MCL1 | myeloid cell leukemia sequence 1 (BCL2-related) (MCL1), transcript variant 1, mRNA. | 2.05 | 0.20 | 1.49 | 0.06 | NM_021960.3 |
| NEDD9 | neural precursor cell expressed, developmentally down-regulated 9 (NEDD9), transcript variant 1, mRNA. | 2.05 | 0.10 | 1.45 | 0.13 | NM_006403.2 |
| CTGF | connective tissue growth factor (CTGF), mRNA. | 2.04 | 0.07 | 1.59 | 0.46 | NM_001901.1 |
| PER2 | period homolog 2 (Drosophila) (PER2), transcript variant 1, mRNA. | 2.04 | 0.30 | 1.45 | 0.19 | NM_022817.1 |
| KIAA1754 | KIAA1754 (KIAA1754), mRNA. | 2.03 | 0.10 | 1.66 | 0.14 | NM_033397.2 |
| C1orf106 | chromosome 1 open reading frame 106 (C1orf106), mRNA. | 2.01 | 0.27 | 1.45 | 0.94 | NM_018265.1 |
| RGC32 | response gene to complement 32 (RGC32), mRNA. | 2.00 | 0.60 | 1.77 | 0.15 | NM_014059.1 |

Micaroarray assay was done twice and data were represented as average value ± S.D. as of induction fold.

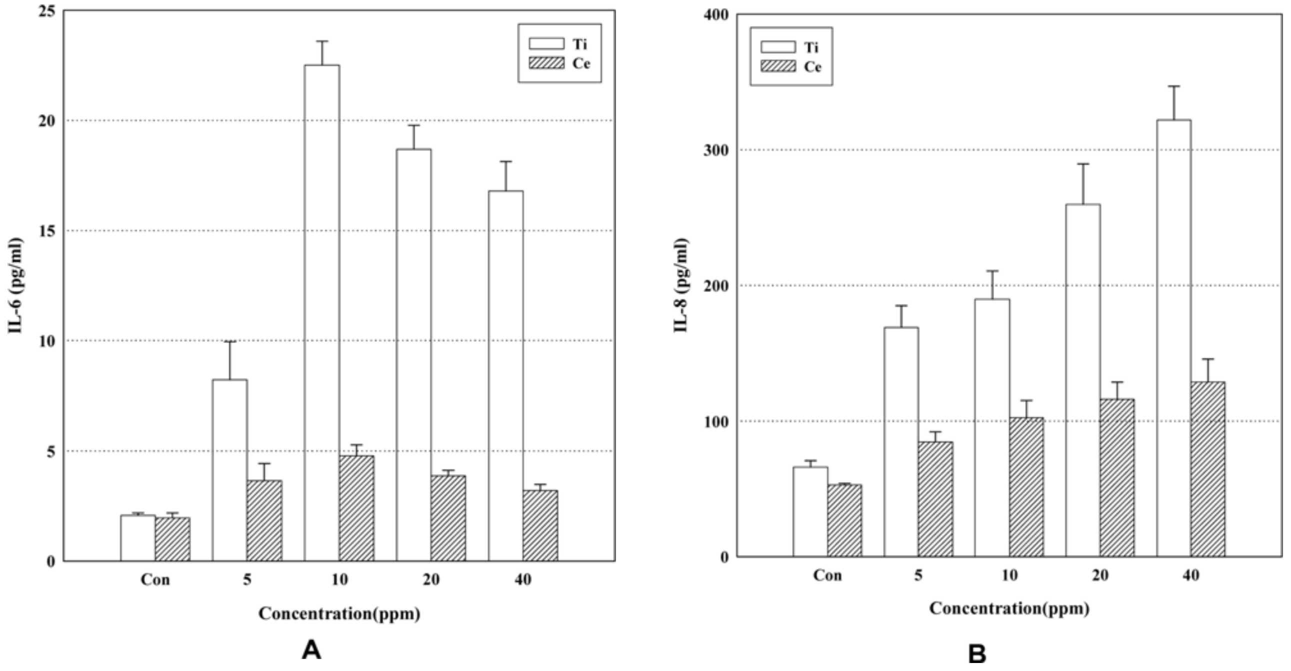

**Fig. 4.** Increased level of secreted cytokines (IL-6 and IL-8) in TNP- and CNP-treated cells. Cells were treated with TNP and CNP with the designated concentrations for 24 hours. Cytokines were measured in the supernatant using ELISA kits. **A**: IL-6, **B**: IL-8. All treated group showed statistical differences from non-treated control group.

information materials from nucleus. Furthermore, inhibition of transcription by interaction between nanoparticles transcriptional machinery proteins may be another reason (Walther *et al.*, 2002). However, no evidence has been reported at present time.

Microarray data on the gene expressions in cultured BEAS-2B cells treated with TNP and CNP, were re-confirmed by the measurement of secreted cytokine level, IL-6 and IL-8 (Fig. 4). As shown in Fig.4A, secreted IL-6 levels were increased in a dose-dependent manner and showed maximum level (22.5 pg/ml) in TNP 10 ppm-treated cells and then the level was decreased with the increase of concentration. CNP also increased the secreted IL-6 levels and the dose-dependency was very similar to that of TNP. IL-6 level reached maximum level to 4.8 pg/ml in CNP 10 ppm-treated cells. IL-8 which belongs to a family of chemokines, was also increased in TNP- and CNP-treated cells with concentration-dependency. However, the concentration-dependency was different from that of IL-6. Maximal increase of IL-8 (TNP; 321.9 pg/ml, CNP; 128.6 pg/ml) was observed in 40 ppm treatment while maximal increase of IL-6 was shown by 10 ppm treatment (Fig. 4B). TNP was more potent than CNP in the gene induction with all ranges of exposure concentrations, which is the same as the result of microarray analysis (Table 1).

***Correlation between the induction levels of genes by TNP and CNP.*** The induction levels of genes by TNP and CNP were compared and plotted for the correlation. Correlation coefficiency was obtained by SPSS. When the induction levels were plotted, it was found that the most genes induced by TNP were also induced by CNP and the induction level of each gene was found to be correlated ($r = 0.645$, $p < 0.01$). The correlation plot is shown in Fig. 5.

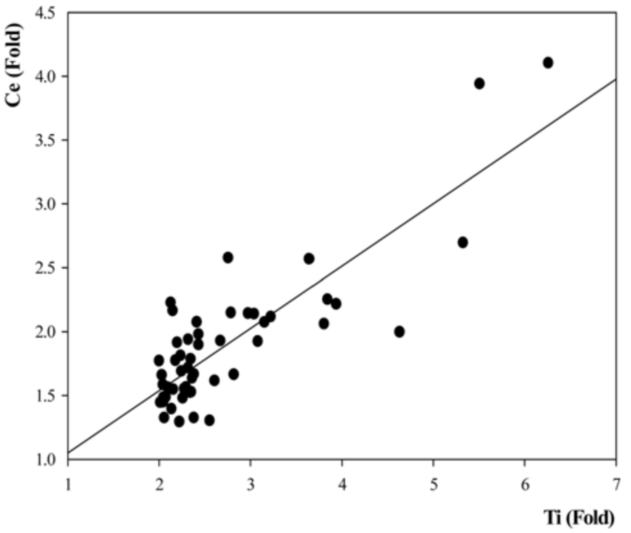

**Fig. 5.** Regressional analysis for correlations of the gene induction levels in TNP- and CNP-treated cells. Correlation value (r value) obtained by SPSS was 0.645. The continuous lines represent the regression lines ($r^2 = 0.70$).

Our previous study showed that TNP exerted cytotoxicity, induced ROS generation, decreased intracellular GSH level, and also induced the expressions of oxidative stress-related genes or inflammation related genes. Cytosolic caspase-3 activation and chromatin condensation were also shown in the TNP-treated cells, which suggested the apoptotic process (Park *et al.*, 2008a, b). Very similar toxic responses were also observed in CNP-treated BEAS-2B cells. CNP induced ROS generation, decreased intracellular GSH level, induced cytosolic caspase-3 activation and chromatin condensation. Both TNP and CNP penetrated into the plasma membrane and located in the peri-region of nuclear membranes, which may be possible for the nanoparticles to block nuclear pore to inhibit free transport of information molecules from nucleus to cytoplasm (from cytoplasm to nucleus).

We showed close correlations of induction levels of genes between TNP- and CNP-treated cultured cells using microarray analysis in this study. This may be contributable to establish a reasonable idea to explain possible common toxic mechanism of metal oxide nanoparticles.
